# Schiff base metal complexes as emerging therapeutics against antimicrobial-resistant skin pathogens

**DOI:** 10.5599/admet.3214

**Published:** 2026-03-06

**Authors:** Parami Sinhapitiya, Samawansha Tennakoon, Isuri A. D. K. Weeraratne

**Affiliations:** 1 Department of Chemistry, Faculty of Applied Sciences, University of Sri Jayewardenepura, Sri Lanka; 2 Genetics and Molecular Biology Unit, Faculty of Applied Sciences, University of Sri Jayewardenepura, Sri Lanka

**Keywords:** Skin infections, minimum inhibitory concentration, antifungal agents, antibacterial agents

## Abstract

**Background and purpose:**

The development of antimicrobial resistance reduces the efficacy of antimicrobial agents and poses a significant challenge to treat skin diseases. Many scientists, researchers, and pharmaceutical companies work diligently to investigate novel antimicrobial agents and discover alternatives to existing ones, aiming to address antimicrobial resistance. Within the broad field of metal complexes, Schiff base complexes occupy a prominent position, with structural versatility and significant biological properties that make them promising candidates for developing alternative drugs to combat the global crisis of antimicrobial resistance.

**Experimental approach:**

This paper reviewed the existing literature on how the structural features of some recently studied Schiff base ligands and their complexes influence the antibacterial and antifungal activities of these compounds against common skin pathogens, including *Candida albicans* sp., dermophytes, *Staphylococcus aureus* and *Streptococcus pyogenes*.

**Key results:**

The structural features, including the azomethine group (C=N), heteroatoms and substituents, in Schiff base compounds have been associated with interference with protein synthesis and the growth of bacterial and fungal cells. Schiff base compounds affect cell wall and cell membrane synthesis and inhibit enzymes essential to cell division and other cellular mechanisms. The chelation theory and the overtone’s concept suggest that Schiff base metal complexes exhibit higher antibacterial and antifungal activities compared to Schiff base ligands.

**Conclusion:**

This review focuses on providing an overview of how the structural features of Schiff base compounds influence the antimicrobial properties of these compounds against *Candida albicans* sp., dermophytes, S*taphylococcus aureus* and *Streptococcus pyogenes*.

## Introduction

In the 21^st^ century, antimicrobial resistance (AMR) has become a significant global issue that requires mandatory action. Antimicrobial resistance occurs when bacteria, fungi, viruses and parasites no longer respond to antimicrobial agents [1,2]. Microbial evolution, genetic mutations and transmission of antimicrobial resistant genes have a significant influence on the prevalence of AMR worldwide [3-5]. Antimicrobial resistance poses a daunting challenge in medicine by rendering the effectiveness of medications available for the treatment of infections.

The skin accounts for 10 to 16 % of total body mass and is susceptible to bacteria, fungi, viruses, and protozoa [6-9]. Acne, alopecia, decubitus ulcers, pruritus, psoriasis, scabies, urticaria, bacterial, fungal, and viral skin diseases are among the common skin diseases [10-12]. Skin disorders have exhibited detrimental impacts on physical and mental health, quality of life, national health systems, the economy of a country and high mortality rates [13-15]. Approximately 4.9 billion new fungal, bacterial, skin and subcutaneous diseases were reported worldwide [16]. According to the Global Burden of Disease analysis, bacterial skin infections accounted for 76,000 deaths in 2017 [17-19]. Over the past two decades, the rise in invasive fungal infections has posed significant challenges to various fields, including healthcare, medicine, public health, and the global economy and based on past literature, approximately 300 million people are affected by fungal infections each year [20,21]. Treating bacterial and fungal skin infections has become of prime importance worldwide due to the emergence and rapid spread of antimicrobial resistance. With advances in antibiotics, antifungal agents, and other therapeutic agents, many medications are available to treat skin diseases. However, antimicrobial resistance hinders the efficiency of these treatments. Thus, there is an urgent need to explore novel therapeutic approaches to overcome this global crisis [22,23].

Antimicrobial peptides, antivirulence drugs, phage therapy, vaccination methods, plant-derived extracts, metal nanoparticles and metal complex-based antimicrobial compounds are among recent approaches to combat AMR [24-27]. However, the chemical properties of metal complexes, including the diversity of metals and ligands, oxidation states, three-dimensional geometries and physical properties, make them ideal candidates for the development of structurally diverse antimicrobial drugs [28-31]. Furthermore, the ability of metal complexes to inhibit cellular functions and metabolism is essential for the development of novel antimicrobial agents [30]. Among various types of metal complexes, Schiff base complexes have gained much attention in the scientific community due to their chemical, physical and biological properties. Previous studies have shown a range of biological properties of Schiff base metal complexes, including antibacterial, antifungal, antioxidant, anticancer, anti-inflammatory, antimalarial and antiviral properties [29,31-35].

This review provides an overview of how structural features influence the antibacterial and antifungal activities of recently studied Schiff base ligands and their complexes against common bacterial (Staph*ylococcus* aureus, *Streptococcus pyogenes*) and fungal (*Candida albicans*, dermophytes) skin pathogens, based on MIC values.

## Antimicrobial resistance

The evolution of resistance in bacteria, fungi, viruses, and parasites to antimicrobial drugs, known as antimicrobial resistance (AMR), poses a formidable challenge to treat microbial infections.

The main drivers of antimicrobial resistance (AMR) fall into different categories, including human-influenced, environmental and animal-associated factors [36,37]. Inappropriate and excessive use of antimicrobial agents are primary cause of antimicrobial resistance worldwide [36]. These primary causes subject microbes to selective pressure, driving the development of resistance within them for long-term environmental survival [38,39]. In addition, human migration, the export and import of goods, lack of knowledge about antimicrobial agents, low standards for antimicrobial agent production and improper storage of drugs could promote the spread of AMR globally. Moreover, the use of antimicrobial drugs in farm animals to treat and prevent various illnesses can facilitate the spread of AMR to humans through animal consumption [40].

### Mechanisms of resistance of bacteria and fungi against antimicrobial agents

Bacteria and other microorganisms confer resistance to antimicrobial agents through mutations and the horizontal transfer of resistance genes and adaptive genetic elements [40]. In addition to resistance acquired through mutations and horizontal gene transfer, various mechanisms within bacterial cells confer resistance to antimicrobial agents.

The outer barrier in bacterial cells prevents the penetration of toxic substances into the cell and this process is different in Gram-negative and Gram-positive bacteria based on the distinct features of their cell walls [41,42]. Microbes develop resistance by altering their cell walls, which contain target sites for antimicrobial agents [40,43]. Mutations and shrinkage of the cell membrane porin channels alter the permeability of the cell membrane and restrict the entry of antimicrobial drugs into bacterial cells [44,45]. Moreover, the ability of bacterial cells to form biofilms protects them from antimicrobial agents [46]. The presence of antimicrobial agents at the bacterial cell site affects the function of these drugs by reducing drug uptake and activating efflux mechanisms in bacterial cells [4,5]. Furthermore, transport proteins in resistant microbial cells counteract antimicrobial agents by pumping them out of the cell into the external environment via efflux pump mechanisms [46,47]. Bacterial cells contain genes that encode enzymes, including lyases, hydrolases and transferases and they are involved in modifying, inactivating or rendering the activity of antimicrobial drugs [48]. β-lactam antibiotics inhibit cell wall synthesis by binding to proteins in bacterial cell membranes. Bacterial cells contain β-lactamase enzymes coding genes that hydrolyse the β-lactam rings and affect the activity of β-lactam [49,50]. Fungal cells have also remodelled their cell walls, altered the polysaccharide content of their cell wall and genetically adapted to resist antifungal agents [51].

## Skin structure and functions

**Figure 1.**
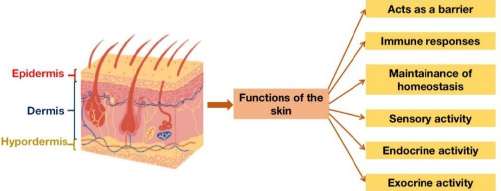
Structure of the skin and its functions

Skin, being the largest organ in the body, acts as the interface between an organism and its external environment and enabling interactions with the surrounding environment [52]. The skin is a multi-layered structure consisting of three main differentiated layers: epidermis, dermis and hypodermis [53-55]. Skin can perform numerous functions, as shown in Figure 1 [56-58]. Skin provides protection against harmful external factors, including chemicals, radiation, virulent pathogens and allergens [58,59]. In addition, the skin helps regulate body temperature [60,61]. Furthermore, the skin produces a variety of hormones, sex steroids, melatonin and vitamin D, which are crucial for body functions [57-59,62].

## Skin diseases and infections

Skin diseases are recognized as the fourth leading cause of human illnesses globally, contributing to the Global Disease Burden (GDB) [63-65]. Past studies have shown that skin disorders affect 30 to 70 % of individuals worldwide [66,67]. The invasion and proliferation of bacteria, fungi, viruses and parasites on the skin lead to skin infections and are classified into two types: primary and secondary infections [2]. Skin infections spread worldwide due to low public awareness regarding the types of skin infections, their symptoms, the inappropriate use of antimicrobial agents as treatments, as well as sharing of belongings, tattooing and piercing [68-71]. Although most skin-residing pathogens are commensals, some factors, including host genetic variation, microbial dysbiosis and immune status, can convert commensals into a pathogenic state [72].

## Bacterial pathogens causing common skin infections

Bacterial dermatological disorders are among the leading health problems worldwide. Data used by Gu *et al*. [63] in 2025 on Global Disease Burden by bacteria from 1990 to 2045 estimated a possible increase in incidents by 2045. Previous studies have shown that bacterial skin infections rank as the 28^th^ most prevalent disease among hospitalized patients [73]. Globally, the most common bacterial pathogens responsible for skin infections are Gram-positive *Staphylococcus aureus* and *Streptococcus pyogenes*.

### Staphylococcus aureus

*Staphylococcus aureus* is a coccal-shaped bacterium that resides on the skin and mucous membrane of healthy individuals [74,75]. This microorganism can produce various toxins, such as Panton-Valentine Leucocidin (PVL), exfoliatins (ETs), enterotoxins, and toxic shock syndrome toxin 1 (TSST-1), which cause various dermatological manifestations regardless of age and geographical distribution [76-78]. In addition to skin diseases, this microbe causes septicemia, pneumonia, ocular infections, and central nervous system infections [79]. Some common dermatological infections caused by *Staphylococcus aureus* are tabulated in Table 1.

**Table 1.** Frequently observed bacterial infections caused by *Staphylococcus aureus* and *Streptococcus pyogenes*

Bacterial disease	Causative agent(s)	Target organs	Symptoms	Reference(s)
Impetigo	*Staphylococcus aureus* and/or *Streptococcus pyogenes*	Mouth (infants), skin	Erythematous papules, pustules, bullae, honey-coloured scabs and crusts	[76,77,88-90]
Folliculitis	*Staphylococcus aureus*	Thighs, perineum, arms, eyelid, scalp, trunk, arms and legs	Multiple/ single lesions on densely haired parts	[73,76,79,91,92]
Abscesses	*Staphylococcus aureus*	Face, chest, lower abdomen, buttocks underarms and groin	Erythematous plaques, red and painful pus	[76,77,93]
Cellulitis	*Streptococcus pyogenes* or *Staphylococcus aureus*	Legs, feet, arms, hands and face	Acute, painful swelling and redness on the skin	[73,94]
Erysipelas	*Streptococcus pyogenes*	Legs and face	Lymphatic streaking and erythematous with margins	[73]
Furuncles	*Staphylococcus aureus*	Armpits and gluteal region	Red, painful nodules with pustules	[77,95]

### Streptococcus pyogenes

*Streptococcus pyogenes*, also known as Group A *Streptococcus* (GAS), is a coccus pathogen that causes a variety of infections in the respiratory tract and skin [80,81]. This pathogen mainly targets young adults and small children who are living in underprivileged countries, while GAS diseases remain endemic in economically developed countries, including the United States of America and Australia [82-84]. Pathogenicity of *Streptococcus pyogenes* is mediated by virulence factors, including pili, M proteins, leukocidins, streptolysins, immunoglobulin-degrading enzymes and superantigens [85]. A study performed in 2005 indicated that 18.1 million people are affected by the global burden of GAS and 1.78 million new cases occur each year [86]. According to the World Health Organization (WHO), GAS is estimated to be the ninth leading cause of death in humans [87]. Frequently observed infections caused by *Streptococcus pyogenes* are summarized in Table 1.

## Fungal pathogens causing common skin infections

Being globally widespread, fungi play a significant role in the environment, humans and plants [96,97]. According to Havlickova *et al*. [98], approximately 20 to 25 % of the world population is prone to fungal skin infections. The most common groups of fungi that cause superficial fungal infections are dermatophytes, molds and yeasts [97,99-101]. Among these three groups, *Candida* sp. is the most common skin-infecting fungal pathogen belonging to the yeast group [102].

### Candida spp.

*Candida* species are identified as the most frequent human fungal pathogens that inhabit distinct areas within the host [103,104]. *C*. *albicans* can colonize on the skin, genital and intestinal mucosa of approximately 70 % of healthy individuals [105]. Moreover, a survey conducted at an international autopsy program for leukaemia patients indicated that *Candida* sp. cause 58 % of fungal infections [106]. Some common skin infections caused by *Candida* sp. and their symptoms are summarized in Table 2.

**Table 2.** Frequently observed fungal infections caused by *Candida* spp. and Dermophytes

Fungal disease	Causative agent(s)	Target organ(s)	Symptoms	Reference(s)
Tinea pedis	*Trichophyton rubrum*, *Trichophyton interdigitale*	Feet, leg	Peeling and irritation between the toes	[112]
Tinea capitis	*Trichophyton tonsurans, Microsporum audouinii*	Scalp	Hair loss in scalp, weeping, irritating, crusty lesions	[113-116]
Onychomycosis	*Trichophyton rubrum, Trichophyton mentagrophytes*, *Candida* spp., some molds	Nails	Stiff, faded broken nails	[117,118]
Tinea versicolor	*Malassezia furfur*	Chest, neck and arms	Painful red scaly lesions	[119]
Tinea corporis	*Trichophyton rubrum, Trichophyton tonsurans, Microsporum canis*	Entire body (skin)	Scaly painful red plaques and annular lesions with edges, overlapped lesions	[114,120]
Mucosal, oral and genital candidiasis	*Candida* spp. (*Candida albicans*)	Tongue, genitals	Itching, erythematous, white plaques on cheeks	[121-123]
Chronic mucocutaneous candidiasis	*Candida* spp. *(Candida albicans)*	Face, neck, trunk and nails.	White grooved lesions, scaly, nodular and moist lesions	[124,125]
Invasive candidiasis/ candidaemia	*Candida* spp.	Mouth, genitals, eyes, kidneys, liver, and brain	Continued fever, losing weight, abdominal pain and swelling of the liver and spleen	[126]

### Dermophytes

Dermophytes are a category of co-related ascomycete filamentous fungi that infect tissues including skin, hair and nails [107]. According to Achterman *et al*. [108], in the USA half a billion dollars is spent annually on medication for dermophyte infections. The WHO has estimated that the global prevalence of dermatomycosis is approximately 20 % [109]. In a healthy population, dermatophytosis is the fourth leading cause of global burden, with a predicted prevalence of 20-25% [110]. Superficial infections caused by dermatophytes, such as *Epidermophyton*, *Microsporum* and *Trichophyton* affect 1.7 billion people worldwide [111]. Some frequently observed fungal infections caused by dermophytes are shown in Table 2.

## Importance of Schiff base compounds in combating antimicrobial resistance

Widespread AMR poses a threat to patients with skin infections and highly contributes to increased mortality rates. Unless proper action against this silent pandemic is taken, AMR will be the major cause of death in the future, and by 2050, approximately the number of deaths due to AMR will rise to 10 million [22]. Consequently, AMR has necessitated the development of novel therapeutic agents to address this global burden.

In modern science, one breakthrough is the discovery and development of antimicrobial agents to suppress infectious diseases, which are harmful to health and the global economy [127]. Schiff bases and their metal complexes exhibit a wide range of biological properties, including antitumor [128], anticancer [129], antibacterial [130], antifungal [131], anti-inflammatory [132], antimalarial, antiviral activity [133], antiparasitic, antiproliferative, antioxidant, anti-tuberculosis [134], antipyretic, anti-HIV and anticonvulsant [133], which could be useful and applicable in many scientific disciplines. These diverse biological properties encourage scientists to explore new metal-based drugs to overcome the global burden of drug resistance in microorganisms [135]. Advances in inorganic chemistry and scientific investigations into metal-based drugs have led to the use of metal complexes as therapeutic agents for treating human diseases [136].

## Synthesis of Schiff base ligands and metal complexes and their characteristics

Coordination chemistry is a branch of knowledge that covers a wide range from therapeutics to the environment. Schiff base metal complexes play an important role in coordination chemistry and significantly contribute to the advancement of diverse fields of chemistry [137]. After the discovery of Schiff bases in 1864 by Hugo Schiff [138], many researchers have synthesized, characterized, and analysed these compounds and their applications in organic, inorganic, analytical and biological disciplines.

A condensation reaction between a primary amine and either an aldehyde or a ketone in the presence of an acid/base catalyst or under neutral conditions results in a type of organic compound known as Schiff bases with a peculiar feature of an azomethine group (-CH=N-) or an imine group (-C=N-) [139-142]. Nitrogen in the azomethine group (C=N) contains two highly reactive pairs of electrons involved in Schiff base complex formation, while the carbon is liable to nucleophilic addition [143]. In the first step of the condensation reaction, an unstable carbinolamine is formed, which is dehydrated in the second step [144]. The dehydration step of carbinolamine can be either base or acid-catalysed. In most cases, the dehydration step of carbinolamine is acid-catalysed [145]. Nevertheless, a high hydrogen ion concentration can protonate amines, making them non-nucleophilic and preventing carbinolamine formation. Hence, a suitable mild pH value is recommended for the reaction [145]. Different synthesizing methods of Schiff base ligands include solvent-free synthesis by microwave irradiation, catalyst-based solvent-free synthesis, solvent and catalyst-free synthesis and solvent-based synthesis [146].

The nature of the aldehyde/ketone and the primary amine accounts for the stability of the resulting product [146]. In ketones, the electrophilic nature of the carbonyl group will be reduced by the groups attached to the carbonyl carbon [144]. The low steric hindrance of aldehydes makes them more reactive with primary amines than ketones. Furthermore, compared to aliphatic Schiff bases, aromatic Schiff bases are more efficacious due to the strength of the imine bond [147].

One of the most significant findings in coordination chemistry is the ability of Schiff bases to act as ligands, which has caught the attention of the scientific community. A ligand’s ability to coordinate with a metal ion mainly relies on the identity of the metal, electronegativity and steric factors [145]. The presence of an active imine (-C=N-) group, a lone pair of electrons on nitrogen that can be donated, the basic nature of Schiff bases and a double bond of imine (-C=N-) have made Schiff bases more active and versatile chelating ligands [146,147]. Schiff base ligands can coordinate to various metals, including transition metals, main-group elements, and lanthanides, to form stable complexes [148,149].

During the Schiff base metal complex formation, nonbonding electrons residing on the donor atoms of the Schiff base ligands coordinate with the d orbitals of the metal ions. The lone pair in the sp2 hybridized orbital of the nitrogen atom of the imine (-C=N-) linkage provides the major binding site for metal ions and is responsible for the reactivity, stability, sensitivity, flexibility and broad spectrum of biological properties [150-154]. Interactions between metals and ligands, oxidation numbers and the number of donor atoms of the metal have resulted in elevated biological and pharmacological properties in Schiff base metal complexes [155,156]. The chemical, physical, and biological properties of the resulting Schiff base metal complexes can vary depending on the type of metal bound to the ligand [140].

A thorough study of the nature of metals and ligands, their bonding patterns, stability and the chemistry of metal complexes is essential for researchers to synthesize new ligands and prepare metal complexes with desirable, predictable properties [157]. Knowledge and understanding of coordination compounds have been enhanced through the exploration of new ligands and their coordination to various metals to produce novel metal complexes [139]. Compared with other types of unstable metal complexes, Schiff base metal complexes have attracted considerable attention and have become among the most studied compounds lately. This is mainly due to the structural variability, easy preparation and intriguing biological and pharmaceutical properties obtained upon complexation and their distinct coordination modes [158-160].

**Figure 2.**
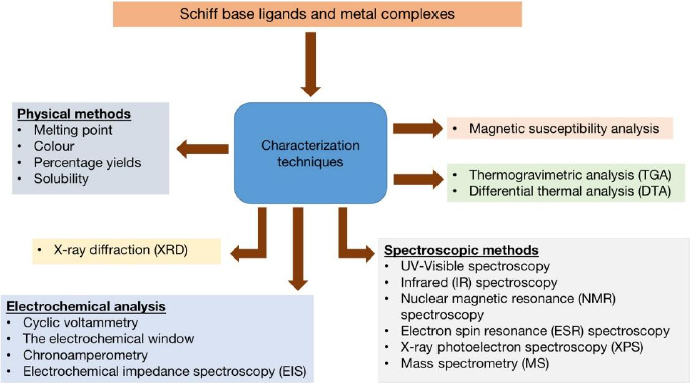
Characterization techniques

After synthesis, the newly synthesized ligands and their complexes must be characterized to gain a thorough understanding of the compounds' functions, purities, properties and structures. Physical, spectral, thermal and elemental (CHN) analyses are among the most widely used analytical techniques [161,162]. In physical analysis, characteristics such as the melting point, colour, yield and solubility will be assessed. During spectral analysis, ultraviolet-visible spectroscopy (UV-Vis), Fourier transform infrared spectroscopy (FTIR), nuclear magnetic resonance spectroscopy (NMR), and mass spectrometry (MS) will be used to confirm the structure [163,164] (Figure 2).

## Mode of action of Schiff base compounds on bacterial and fungal cells

Understanding how the structural features of Schiff base compounds affect microbial cells is important for developing Schiff base ligands and complexes with enhanced antibacterial and antifungal activity.

Schiff bases exhibit antimicrobial activity primarily through the azomethine group (C=N). It forms hydrogen bonds with bacterial cell components and interferes with the cellular protein synthesis [160,165]. Hetero atoms, including oxygen, nitrogen and sulphur, present in Schiff base compounds have also been shown to interact with essential trace elements in bacterial cells and affect growth and function [165]. Moreover, substituents such as electron-withdrawing groups present in Schiff base compounds govern the extent of antimicrobial activity against microorganisms [166]. Carboxyl (COOH) groups present in Schiff base compounds affect the denaturation of protein molecules within bacterial cells [167]. The presence of hydroxyl (OH) groups strongly influences the antimicrobial activity of Schiff base compounds [166]. Ren *et al.* [168] demonstrated that ortho-hydroxyl (o-OH) substituted compound exhibited greater antimicrobial activity compared to para-hydroxyl (p-OH). Furthermore, Hou *et al.* [166] suggested that the charge density distribution in Schiff base compounds strongly contributes to antibacterial activity, whereas steric hindrance of the phenyl ring and partition coefficient factors influence it to a lesser extent.

Much literature has documented the fact that the antimicrobial activity of Schiff base metal complexes is greater than that of the Schiff base ligands. The chelation theory and the overtone’s concept clearly explain why Schiff base metal complexes exhibit greater antimicrobial activity than Schiff base ligands. According to the chelation theory, the positive charge on the metal is reduced and shared with the attached donor groups, delocalizing the π-electrons throughout the chelate ring. As a consequence, the lipophilicity of the central metals will be increased, thereby enhance microbial cell wall penetration and disrupt cellular processes and biochemical reactions in microbial cells [169,170]. According to the overtone’s concept, interactions between metal complexes and the microorganism’s cell membrane are facilitated by the reduction in polarity and positive charge of the metal ion [169].

Not only the structural features of Schiff base compounds, but also those of microbial cells influence antimicrobial activity. Structural variability of bacterial cell walls results in Gram-positive and Gram-negative bacteria [171]. Because the thick peptidoglycan layer of the Gram-positive bacterial cell wall readily absorbs foreign substances, significant antibacterial activity is often observed against Gram-positive bacteria compared to Gram-negative bacteria [171]. Low antibacterial activity against Gram-negative bacteria is mainly due to limited cell wall penetration and efflux pump-mediated removal of Schiff base compounds from bacterial cells [171,172].

Past studies have thoroughly demonstrated the effect of Schiff base compounds on bacterial and fungal cells. To study how Schiff base ligands and their complexes interact with *S. aureus*, Zhang *et al*. [173] conducted an experiment in 2006. In this study, microcalorimetry was employed to characterize bacterial growth and the interaction of Schiff base compounds with bacterial cells [173]. The growth curve results demonstrated the significant growth inhibition in *S. aureus.* Usually, the bacterial growth curve shows two peaks, indicating different metabolic pathways. The study found that one metabolic pathway was more affected than the other at high concentrations of Schiff base compounds [173].

In a separate study, a cellulose-based Schiff base ligand (SCF) and its Cu complex (SCF-Cu) were synthesized. The bacterial apoptosis study revealed the disruption and lysis of most *E. coli* and *S. aureus* cells. Compared to the Schiff base ligand (SCF), treatment with Cu complex (SCF-Cu) exhibited pronounced bacterial cell disruption, cell structural deformation and collapsed surface [174].

The effects of Schiff base complexes on respiration and cell wall components in *Candida* species were investigated by Geweely in 2009 [175]. This study has demonstrated that Schiff base Zn complexes synthesized herein inhibit enzymes responsible for fungal cell wall synthesis. This interfered with the synthesis and assembly of cell wall components, resulting in amino acid leakage [175]. The antifungal activity of the synthesized Schiff base ligand and its metal complexes on *Candida* cell respiration was evaluated by respirometry using Oxygraph. Out of the synthesized complexes, Zn complexes exhibited a reduction in respiration of *Candida* cells, suggesting that Zn(II) complexes interfere with cellular enzymes involved in respiration [175]. According to Coyle *et al*. [176], the function of mitochondria can be affected by transition metal complexes. Metal complexes stimulate the oxidative stress in fungal cells and disrupt the respiratory oxygen uptake of *C*. *albicans* [177].

Another study demonstrated that Schiff bases suppress bacterial growth, based on molecular docking data. The study results revealed that Schiff base compounds showed a greater affinity to the second and third sites of dihydrofolate reductase enzyme in *S. aureus.* This enzyme is responsible for cell division and disruption of this enzyme prevents the synthesis of RNA, DNA and protein. High binding energies further proved the significant growth suppression of *S. aureus* by Schiff base compounds [178].

Another study was conducted to assess the effect of Schiff base compounds on the plasma membrane. This was assessed by extracellular ionic conductivity and the study results revealed that yeast cells treated with nitro group-containing chalcone Schiff bases cause damage to yeast cell membranes, leading to ion leakage [179].

A study conducted by Chung *et al*. [74] demonstrated the potential of the Schiff base Cu complex to enhance bacterial cell wall porosity. Moreover, the study showed how Schiff base Cu complex facilitates the penetration of the Schiff base compound into the cell [180]. In addition, the author suggested that the Schiff base derivatives synthesized in this study may interfere with the biofilm constituents and their biosynthetic pathways of methicillin-resistant *S. aureus* [181,182].

## Schiff bases and their metal complexes as antimicrobial agents

Comprehensive research on Schiff base metal complexes has become a rapidly expanding, multifaceted field of study, contributing to various disciplines of science [183]. The applications of these compounds in chemistry-related fields, including bioinorganic, biomedical, materials, organic and analytical chemistry, continue to attract the attention of many researchers [184-187]. Both Schiff base ligands and their metal complexes have been identified as ideal candidates to cure diseases [157]. Antimicrobial resistance in methicillin-resistant *Staphylococcus aureus* (MRSA), *Staphylococcus aureus*, *Candida* spp. and dermatophytes poses a considerable challenge in managing skin infections caused by these skin pathogens. Numerous recent reports have documented the pronounced antibacterial and antifungal activities of Schiff base compounds against these pathogens, underscoring their therapeutic potential to treat skin infections caused by resistant skin pathogens.

### Antibacterial activity

A series of novel Schiff base complexes was synthesized and screened for antibacterial activity in 2015 by Zafar *et al*. [188] (Table 3, Compound **1**). In this study, the antibacterial and antifungal activities of the novel products and the minimum inhibitory concentrations (MIC) were assessed. Among the prepared complexes, Cu(II), Zn(II), and Co(II) complexes showed the greatest activity in the order of Cu(II) > Zn(II) > Co(II) against *Streptococcus pyogenes.* Among these three complexes, the Cu(II) complex showed significant antibacterial activity, approximately comparable to the reference drug ciprofloxacin (MIC - 6.28 μg/ml) [188]. The oxidative nature of copper-containing Schiff base complexes, greater affinity of Schiff bases towards copper (Cu), greater binding capacity of the metal centre (Cu) and the ability of Cu to damage DNA upon chelation explain the observations of this study [189-193].

**Table 3.** Structures of the compounds

Compound	Structure of the compound(s)	Reference(s)
**1**	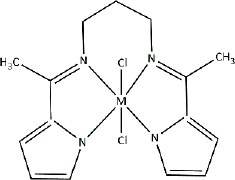	[188]
**2**	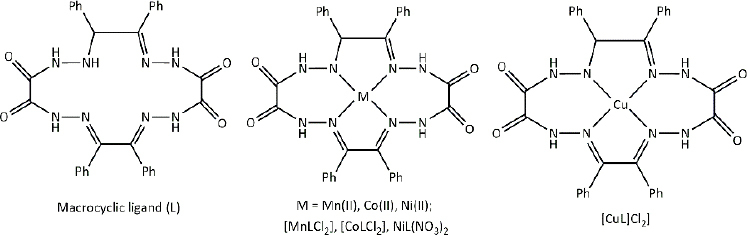	[194]
**3**	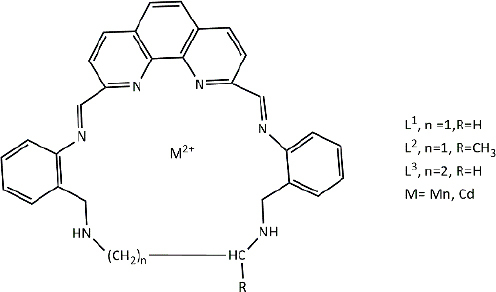	[195]
**4**	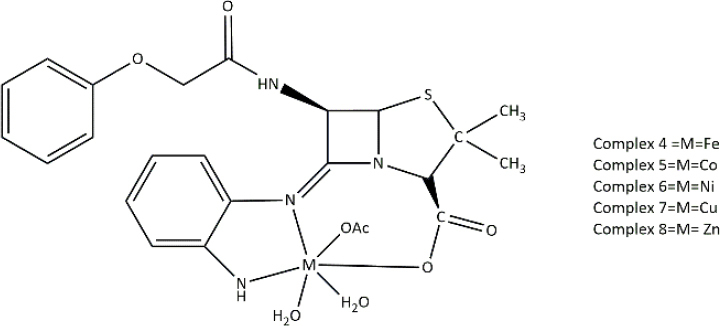	[196]
**5**	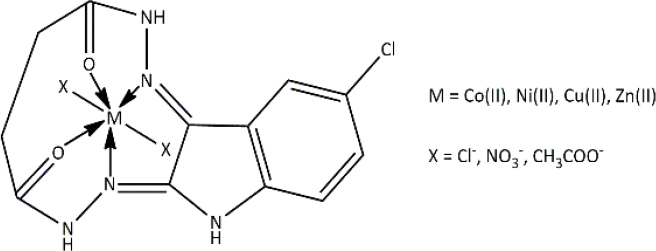	[197]
**6**	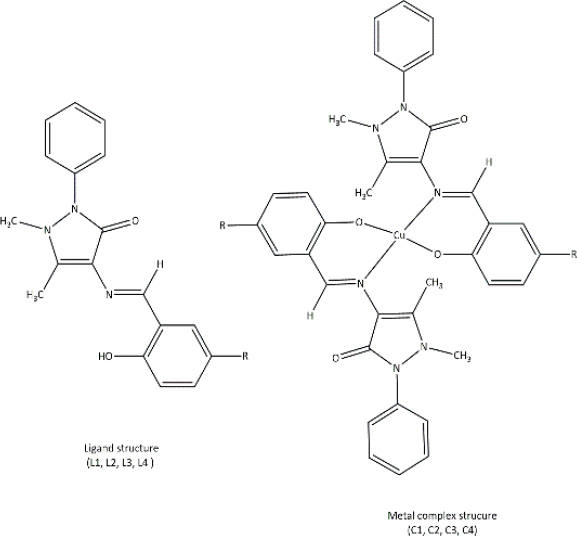	[172]
**7**	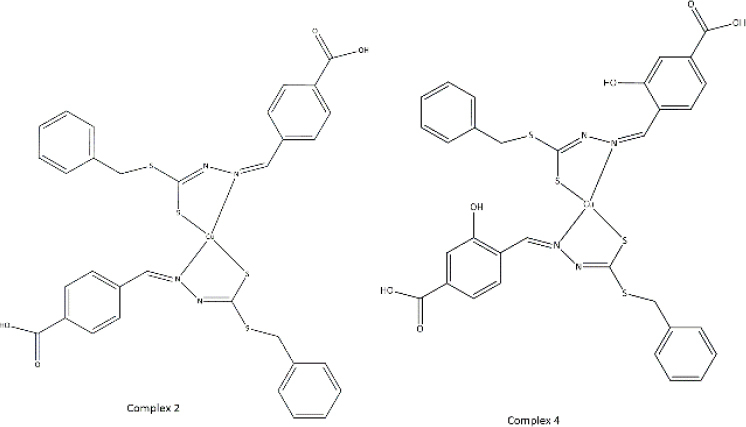	[74]
**8**	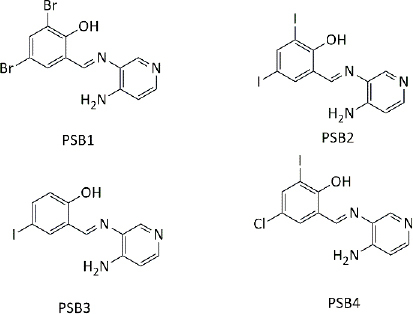	[209]
**9**	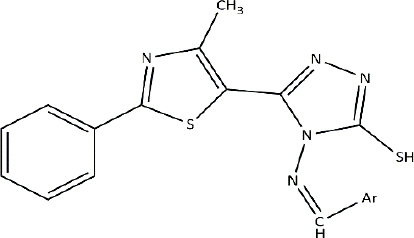	[211]
**10**	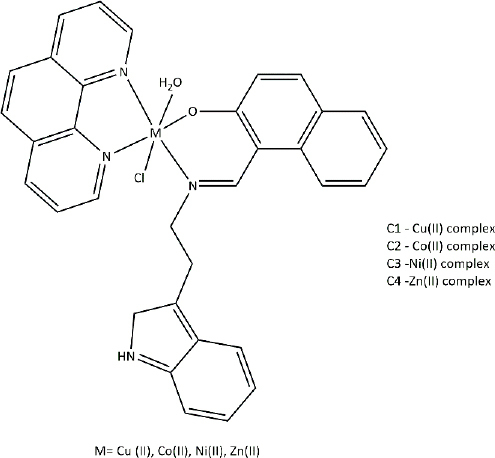	[214]
**11**	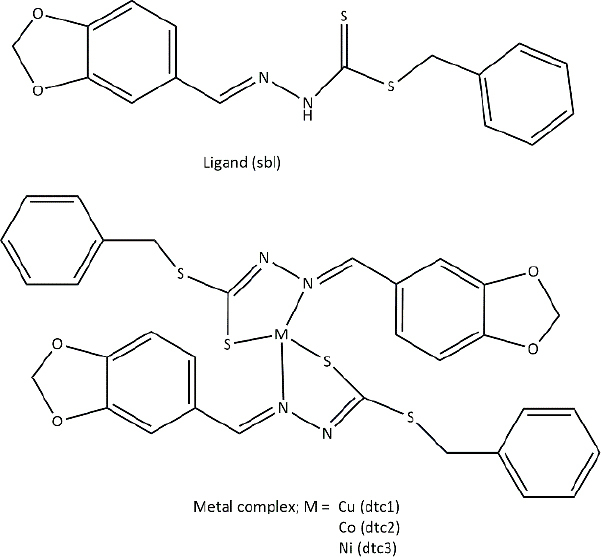	[216]
**12**	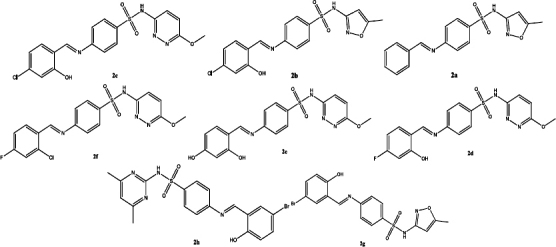	[218]
**13**	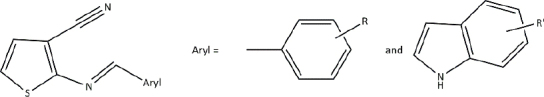	[220]
**14**	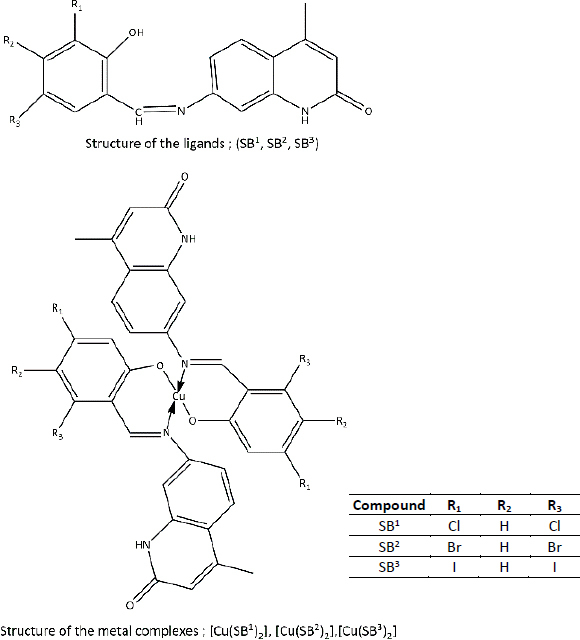	[221]

In the same year, Zafar *et al*. [194] reported another study investigating the biological properties of octaazamacrocyclic ligands and their metal complexes. The structures of the synthesized compounds are tabulated in Table 3, Compound **2**. In terms of antibacterial studies, the zone of inhibition, the minimum inhibitory concentrations (MIC) and the minimum bacterial concentrations (MBC) of the synthesized ligand and the metal complexes were evaluated. According to the results, the ligand demonstrated lower antibacterial activity than the metal complexes. The results of this study can be explained by chelation theory and the concept of overtones [169,170,194]. In the study, *S. aureus* (Gram-positive) showed greater activity than *E. coli* (Gram-negative), which could be explained by differences in the bacterial cell wall [171]. Based on prior literature, counter ions also contribute to more pronounced antimicrobial activity, which may explain the observed effects in this study [156].

In a study by Keypour *et al.* [195], newly synthesized macrocyclic complexes (Table 3, Compound **3**) were evaluated for antibacterial activity using MIC values. Based on the results of this study, more pronounced antibacterial activity was observed against Gram-positive strains compared to Gram-negative strains. Gram-positive bacteria, with thick peptidoglycan layers, can easily absorb foreign substances. Thus, Gram-positive bacteria used in this study may have readily taken up the synthesized novel Schiff base ligands and metal complexes through their cell walls, resulting in significant antibacterial activity [171,172]. These properties have made these compounds important in the production of therapeutic drugs.

Anacona *et al.* [196] described the synthesis of novel Schiff base complexes (Table 3, Compound **4**) and the evaluation of their antibacterial activity. Out of five synthesized complexes, complexes 4 (Fe complex) and 6 (Ni complex) showed the greatest antibacterial activity with an MIC value of 0.042 μmol/ml against methicillin-resistant *Staphylococcus aureus* [196]. Compared with the reference drug's MIC value (0.057 μmol/ml), complexes 4 (Fe complex) and 6 (Ni complex) showed enhanced antibacterial activity, making them ideal for novel drug development [196]. Based on past studies, both iron (Fe) and nickel (Ni) metals and their complexes have exhibited interesting chemical and biological properties, which further describe the results of this study [196].

A study conducted by Ali *et al*. [197] synthesized some macrocyclic complexes (Table 3, Compound **5**) with four metal centres, including Co(II), Ni(II), Cu(II) and Zn(II) and their antibacterial activity was assessed. Based on MIC results, a higher antibacterial activity was observed for [Cu(C_12_H_10_N_5_O_2_Cl)(NO_3_)_2_] and [Zn(C_12_H_10_N_5_O_2_Cl)Cl_2_] complexes with an MIC of 8 μg/mL against *S. aureus*, which is close to the MIC value of the reference drug, ciprofloxacin (MIC - 5 μg/mL) [197]. The presence of Cu as the metal source with diverse biological properties, the ability to cleave DNA upon complex formation and the generation of radicals via the redox reaction between Cu(II) and Cu(I) may account for the enhanced antimicrobial properties observed in this study [198-201]. Moreover, the electronic properties, structural arrangement and geometry and growth inhibitor activities of Zn ion, further support the observations of this study [202-204].

In 2021, Kargar *et al.* [172] synthesized a range of novel copper complexes (Table 3, Compound **6**). Across all tested compounds, metal complexes showed greater antibacterial activity against Gram-positive *S. aureus* than the free ligands, consistent with chelation theory and the overtone’s concept in prior literature [169,170,205,206]. According to Kargar *et al*. [172], these results were further explained by the nature of the bacterial cell wall. Moreover, the presence of a strong electron acceptor in the C4 complex, the presence of an uncoordinated electron-withdrawing nitro group at the para position of the phenyl ring and the nature of the metal centre (Cu) resulted in greater antibacterial activity, which was further validated by studies with similar results [172,199,207].

In 2021, Chung *et al*. [74] reported the synthesis of two copper complexes (Table 3, Compound **7**) and their antibacterial activity was determined against *S. aureus* and methicillin-resistant *S. aureus* (MRSA), via MIC and MBC studies. Among the two complexes, complex 4 exhibited the greatest antibacterial activity against *S. aureus*, which could be a result of the presence of an additional OH group in complex 4 [74]. Based on past studies, OH groups are known to enhance the antibacterial activity against a broad spectrum of bacteria [208]. Complex 2 exhibited the greatest antibacterial activity against methicillin-resistant *S. aureus* compared to the reference drug, oxacillin (OXA), which makes it ideal for clinical use. Factors such as the presence of the COOH group, uncoordinated heteroatoms (nitrogen, sulphur and oxygen), complexation with copper, redox properties posed by the Cu ion, their affinity to biological molecules present in bacterial cells and chelation theory define the observed activity of these compounds against the tested bacterial strains precisely [74,160,165].

According to a study done by Carreño *et al*. [209] in 2024, four pyridine Schiff bases were synthesized (Table 3, Compound **8**). Overall, the PBS2 compound showed the most significant antibacterial activity against all the tested strains. The presence of two iodine substitutes, along with their electronic and steric properties, accounts for the significant antibacterial activity reported in [209]. The presence of the OH group in the compounds facilitates hydrogen bonding with bacterial membranes and components, contributing to pronounced antibacterial activity [210]. Moreover, the electron-donating nature of the OH moiety results in greater electron density in the C=N group, which in turn increases the ability of the ligand to form metal complexes [167]. These properties make these compounds ideal for synthesizing therapeutic compounds with antimicrobial properties.

### Antifungal activity

Thiazoles are chemical compounds with important biological properties. A study by Stana *et al*. [211] in 2016 reported the antifungal activity of the novel thiazolyl-triazole-based Schiff base compounds (Table 3, Compound **9**) against *Candida* species. The presence of electron-withdrawing groups on compounds B5 (-Br) and B10 (-NO2) led to significant antifungal activity, as further supported by a study by [212]. The presence of an uncoordinated electron-withdrawing nitro group in the phenyl ring and the nature of the metal ion influence the microbial growth inhibition [213]. These results were further validated by studies with similar results [134,172,199,207].

A series of new metal complexes was synthesized in a study described by Dar *et al*. [214] in 2019 (Table 3, Compound **10**). All these compounds were assessed for their antifungal activity against fluconazole-resistant and susceptible *Candida* species [214]. C3 (Ni complex) showed the highest anti-*Candida* activity against all the tested C*andida* species and exhibited a greater antifungal activity compared to the reference drug [214,215]. These observations could be explained by chelation theory and the overtone’s concept [214]. The formation of a hydrogen bond between the nitrogen atom of the azomethine (C=N) group and active sites in the cell interrupts cell wall production and disrupts normal cellular processes [169].

Another study by Malik *et al*. [216] reported in 2020 developed s-benzyldithiocarbazate-derived Schiff base ligands and their complexes (Table 3, Compound **11**). Their antifungal activity was screened against ten fluconazole (FLC) susceptible and five resistant *Candida* strains [216]. Study results revealed that the antifungal activity of metal complexes is higher than that of the ligand [214,216]. In past literature, this was explained by chelation theory and overtone’s concept [217]. Nickel (dtc3) complexes exhibited greater antifungal activity, comparable to that of the standard drug (fluconazole). According to Ejidike and Ajibade [169], nickel complexes can penetrate into the microbial cell and disrupt cellular processes upon coordination with Schiff base ligands, which further explains the results of this study.

Another study conducted by Hamad *et al*. [218] in 2021 synthesized sulphonamide-based Schiff bases (Table 3, Compound **12**) and their antifungal activity was screened against *C. albicans*, *C. auris*, *C. glabrata*, C. *krusei*, *C. tropicalis* and *C. parapsilosis.* Compound 2b exhibited the greatest antifungal activity against *C. auris* [218]. The presence of OH and Cl groups in this compound clarifies the observed activity [195]. The presence of the electron-withdrawing chlorine (Cl) group at 2b and the presence of a heteroaryl group on the compound 2c explain the significant fungal activity against *C. auris* [218,219].

With the aim of synthesizing compounds with significant antifungal activity to combat the emergence of resistance in dermophytes, a study was carried out against *Epidermophyton floccosum, Trichophyton tonsurans, Trichophyton mentagrophytes, Trichophyton rubrum and Candida parapsilosis* by Luna *et al*. [220]. In this study, seventeen Schiff bases (Table 3, Compound **13**) were synthesized and their biological activity, antifungal activity, and cytotoxicity were analysed. Overall, greater antifungal activity was observed in compounds containing electron-withdrawing groups, whereas lower antifungal activity was observed in compounds with electron-releasing groups, as supported by numerous studies [172,199,219]. Similar antifungal activity to standard drugs and greater antifungal activity of these compounds of this study are ideal for antimicrobial drug development.

Quinoline-based compounds are well known for their biological properties and have been used in the preparation of therapeutic drugs. A study conducted by Muhammad *et al.* [221] in 2022 synthesized novel quinoline-based compounds and investigated their antifungal activity against four *Candida albicans* strains. The structures of the compounds are shown in Table 3, Compound **14**. This study showed that metal complexes displayed greater antifungal activity than the corresponding ligands against the tested strains, which was theoretically explained by chelation theory and Overton’s concept in many studies [222]. Among the three ligands, SB^1^ showed the highest antifungal activity against all the tested organisms regardless of the type of strain. The presence of the highest electron-withdrawing halogen (Cl) in SB^1^ highly contributes to the remarkable antifungal activity of this complex. This effect has been further proved in several studies [172,199,207,219]. The significant activities of the novel compounds make them promising candidates for the development of novel antifungal agents.

Overall, according to the above-reported studies, the structural features of Schiff base compounds, such as the azomethine group (C=N), the presence and position of OH, COOH groups and uncoordinated electron withdrawing groups (Cl, Br and NO_2_) and the type of metal centre influence the antibacterial and antifungal activities of these compounds. Incorporating these features, or selecting precursors with these structural features, to synthesize novel Schiff base compounds enhances their antimicrobial properties. In the future, this approach will be important in developing novel metal-based drugs to target antimicrobial-resistant skin pathogens.

## Existing challenges

The most common types of obstacles researchers and scientists encounter during Schiff base experiments are solubility, toxicity and stability issues. Solubility issues limit the compound characterization, biological and toxicity studies, thereby limiting the scope of the study [222]. Stability issues pose challenges in handling and storing these compounds for long time periods. Toxicity prevents these compounds from being used in clinical applications [223]. During the experimental level, long reaction times consume lab resources and energy. It hinders the practical application of these compounds in biological and industrial contexts. Although Mishra *et al.* [224] reported the antibacterial potential of Schiff base complexes against skin pathogens in 2022, the stability of these compounds in biological systems remains a research gap, underscoring the need for experimental infection models, pharmacological studies and preclinical testing. Thus, the gap from laboratory studies to biochemical validation hinders the clinical translation of these biologically active compounds. Furthermore, validating the effectiveness of novel antimicrobial drugs in clinical settings is challenging because animal models do not accurately reflect the progression of human diseases [225]. Human physiology makes it more challenging to develop new animal models that accurately represent human diseases [225]. Another challenge is their ability to target a narrow spectrum of microorganisms. Although many scientists are working to develop stronger antimicrobial agents, resistant microbes counter these drugs with sophisticated defence mechanisms, creating an arms race. Past literature documented that AMR cannot be completely eliminated due to microorganisms' ability to survive and adapt to high concentrations of antimicrobial agents, mutations and defence mechanisms [225,226].

### Future outlook

Research studies related to Schiff base synthesis, characterization, biological property evaluation and cytotoxicity have been extensively documented throughout the past years. Schiff base ligands and their complexes in these studies have exhibited pronounced antimicrobial activities, which are ideal for drug production against bacteria, fungi, viruses and parasites. Nevertheless, the clinical applications of these biologically active compounds remain limited and not well established [223]. This poses a challenge for developing novel antimicrobial agents using these compounds to combat antimicrobial resistance.

In most studies on Schiff base compounds, biological activity is determined by measuring the diameters of the zone of inhibition, MIC, MBC and MFC [227]. But more sophisticated techniques must be employed to fully understand the cellular complexity. Moreover, incorporating assays including computational studies (molecular docking and density functional theory (DFT)), biochemical analysis, omic-based characterization and X-ray crystallography would provide more precise knowledge on the interactions of these compounds [209]. It has been documented that omics technologies are important for interpreting molecular targets [74,223]. Since these compounds are candidates for clinical use, evaluating their stability is also mandatory. Switching to novel synthesis methods, such as greener approaches, has led to higher compound yields with less environmental damage, which could be important in future studies [180]. Modification of the Schiff base by incorporating different functional groups could also enhance antimicrobial activity, solubility and penetration through the bacterial membrane [228]. In-depth studies of the relationship between the structure and activity of these compounds are important for developing novel antimicrobial agents for clinical use. In the future, when developing novel antimicrobial agents, attempts should be made to pinpoint the virulence, metabolic and stress response pathways of microbes as target sites of these agents [225].

## Conclusions

In this review, the antibacterial and antifungal activities of some recently synthesized Schiff bases and their complexes were compared with standard drugs using their MIC values against skin infection-causing pathogens, including *Candida albicans* sp., dermophytes, *Staphylococcus aureus* and *Streptococcus pryogens.*

Many studies have shown the remarkable antimicrobial activity of Schiff base ligands and complexes driven by their structural properties and variability. After studying the stability, cytotoxicity and side effects of novel Schiff base complexes, they could be ideal to be utilized as antimicrobial agents and have shown significant contribution to the field of therapeutics. According to previous literature, numerous studies have investigated the antibacterial or antifungal activities of novel Schiff base complexes compared with existing antimicrobial agents. Nevertheless, quite a few studies are continuing through the drug development process. Although many alternative drugs have been synthesized recently, the overuse and inappropriate use of antimicrobial drugs, as well as gene transfer, have led antimicrobial resistance, an enduring and challenging global issue. Thus, in addition to novel drug development, greater emphasis should be placed on linking lab studies to drug-design applications after evaluating the toxicity of newly synthesized compounds. Moreover, it is important to have a thorough understanding of how the antibacterial and antifungal properties of Schiff base complexes vary with their structural properties when synthesizing novel comp compounds that are identified as ideal candidates for therapeutic drug designing.

## Abbreviations

AMR: Antimicrobial resistance
MIC: Minimum inhibitory concentration
MFC: Minimum fungicidal concentration
FTIR: Fourier transform infrared spectroscopy
MS: Mass spectrometry
WHO: World Health Organization
MBC: Minimum bacterial concentration
UV-Vis: Ultraviolet-visible spectroscopy
NMR: Nuclear magnetic resonance spectroscopy
